# Modeling bacterial microcompartment architectures for enhanced cyanobacterial carbon fixation

**DOI:** 10.3389/fpls.2024.1346759

**Published:** 2024-02-15

**Authors:** Daniel S. Trettel, Sara L. Pacheco, Asa K. Laskie, C. Raul Gonzalez-Esquer

**Affiliations:** Los Alamos National Laboratory, Bioscience Division, Microbial and Biome Sciences Group, Los Alamos, NM, United States

**Keywords:** carbon fixation, bacterial microcompartments, carboxysome, molecular dynamics, permeation, phase separation

## Abstract

The carboxysome is a bacterial microcompartment (BMC) which plays a central role in the cyanobacterial CO_2_-concentrating mechanism. These proteinaceous structures consist of an outer protein shell that partitions Rubisco and carbonic anhydrase from the rest of the cytosol, thereby providing a favorable microenvironment that enhances carbon fixation. The modular nature of carboxysomal architectures makes them attractive for a variety of biotechnological applications such as carbon capture and utilization. *In silico* approaches, such as molecular dynamics (MD) simulations, can support future carboxysome redesign efforts by providing new spatio-temporal insights on their structure and function beyond *in vivo* experimental limitations. However, specific computational studies on carboxysomes are limited. Fortunately, all BMC (including the carboxysome) are highly structurally conserved which allows for practical inferences to be made between classes. Here, we review simulations on BMC architectures which shed light on (1) permeation events through the shell and (2) assembly pathways. These models predict the biophysical properties surrounding the central pore in BMC-H shell subunits, which in turn dictate the efficiency of substrate diffusion. Meanwhile, simulations on BMC assembly demonstrate that assembly pathway is largely dictated kinetically by cargo interactions while final morphology is dependent on shell factors. Overall, these findings are contextualized within the wider experimental BMC literature and framed within the opportunities for carboxysome redesign for biomanufacturing and enhanced carbon fixation.

## 1 Introduction

Photoautotrophic microbes, such as algae and cyanobacteria, have shown promise as biomanufacturing platforms which can use CO_2_ as their sole carbon source. At the core of this process lies the enzyme ribulose bisphosphate carboxylase/oxygenase (Rubisco), recognized as the most influential and abundant enzyme in our planet’s carbon cycle (Phillips and Milo, 2009; Raven, 2013; Bar-On and Milo, 2019). Rubisco is used by plants, algae, cyanobacteria, and even some non-photosynthetic chemoautotrophs (Andersson and Backlund, 2008) to assimilate nearly 250 billion tons of carbon from the atmosphere every year (Field et al., 1998). Despite its widespread importance, Rubisco is a catalytically inefficient enzyme in today’s oxygen-rich environment, achieving CO_2_ fixation rates on the order of 1-10 s^-1^ (Flamholz et al., 2019; Davidi et al., 2020) while also capable of photorespiration [Rubisco-catalyzed oxygenation of ribulose bisphosphate (Busch, 2020; Savir et al., 2010)]. Carbon assimilation in aquatic organisms is further complicated by the often low availability of dissolved CO_2_ under ambient conditions (Maberly and Gontero, 2017).

To overcome these challenges, certain aquatic microorganisms evolved ornate CO_2_-concentrating mechanisms (CCMs) (Iñiguez et al., 2020; Badger et al., 1998), which consist of inorganic carbon pumps and Rubisco-filled compartments (pyrenoids and carboxysomes) (Badger and Price, 1992) that work by selectively increasing the CO_2_ concentration around Rubisco (Price et al., 1998). Carboxysomes are a part of a larger class of structurally related protein organelles called bacterial microcompartments (BMC). As a class, BMCs are associated with an array of programmable, modular characteristics that can be leveraged to support biomanufacturing and carbon sequestration applications.

The deployment of CCMs as “modules” for CO_2_ fixation has been suggested as a promising target to bolster the productivity of biomanufacturing platforms that utilize CO_2_ as their primary precursor to produce biomass and biofuels/bioproducts. However, heterologous expression and redesign of CCMs requires mechanistic insights elusive to current high resolution experimental methods. To alleviate this limitation, molecular dynamics (MD) simulations are quickly gaining attention for revealing atomic-detailed processes underpinning CCM assembly and function. MD provides spatio-temporal information which can potentially facilitate rational modifications and *in silico* prototyping. This review will present the current state of MD and other computational applications towards studying and redesigning the core of the cyanobacterial CCM, the carboxysome. Since carboxysome-specific simulations are limited, this review draws on and contextualizes the wider experimental BMC literature with implications for their synthetic adaptation for enhanced carbon fixation.

## 2 The cyanobacterial CO_2_-concentrating mechanism

**Figure 1:**
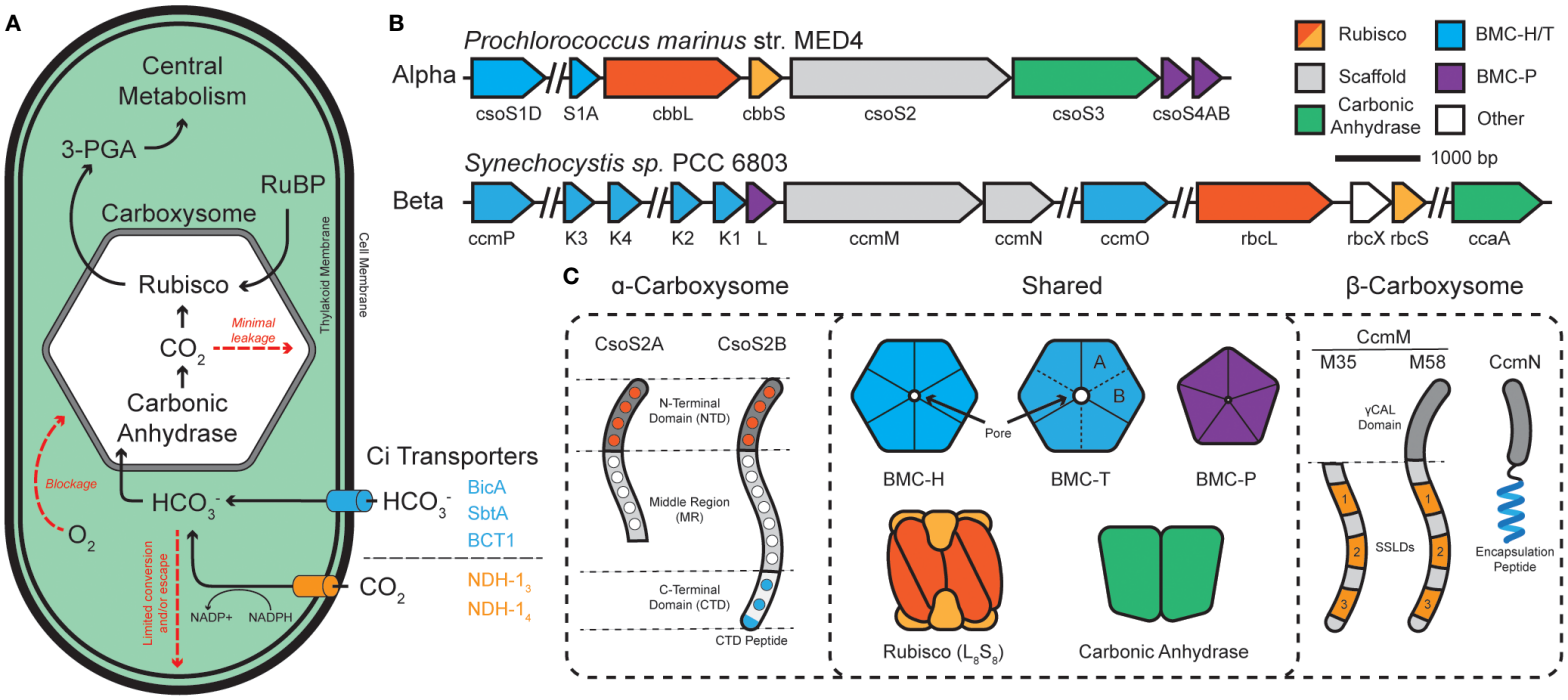
The cyanobacterial carbon concentrating mechanism (CCM) is centered around the carboxysome. **(A)** Cyanobacterial inorganic carbon (C_i_) sequestration begins with CO_2_ and HCO_3_^-^ transporters. Bicarbonate enters the carboxysome and is converted into CO_2_ and combined with ribulose bisphosphate (RuBP) to form central metabolite 3-phosphoglycerate (3-PGA). **(B)** The two classes of carboxysome, ɑ- and β-, differ in their genetic organization. ɑ-carboxysomes tend to organize into distinct operons while β-carboxysomes tend to be more disjointed among numerous satellite loci. **(C)** ɑ- and β-carboxysomes share many components but differ in their use of scaffold proteins. ɑ-types use two forms of CsoS2, which is composed of a multivalent, Rubisco-binding N-terminal domain (NTD; orange dots specify repeats), a multivalent middle region (MR; white dots specify repeats), and a multivalent, shell-binding C-terminal domain (CTD; blue dots specify repeats). CsoS2 is expressed in two forms which differ in their C-termini. β-types use two forms of CcmM to aggregate Rubisco through 3-repeats of small subunit-like domains (SSLDs). The M58 form includes an N-terminal γCAL domain that also binds carbonic anhydrase and CcmN. CcmN contains a C-terminal encapsulation peptide which enables interactions with the shell. Components are colored according to which other components they interact with.

The cyanobacterial CCM has been a focus of multiple studies for the elucidation of structure, function, and its integration into cellular metabolism (Kupriyanova et al., 2023). Carbon assimilation in cyanobacteria begins with the uptake and accumulation of inorganic carbon sources within the cytoplasm (**Figure 1A**). CO_2_ can simply diffuse through the outer cellular membrane while charged bicarbonate must be actively pumped into the cell coupled with Na^+^ or in an ATP-dependent fashion with BicA/SbtA and BCT1, respectively (Shibata et al., 2002a; Shibata et al., 2001; Shibata et al., 2002b). Internalized CO_2_ can be converted to bicarbonate by an NADPH-dependent reduction by the complexes NDH-1_3_ and NDH-1_4_, which are coupled to CO_2_-uptake proteins (Cup) (Artier et al., 2018). CO_2_ and the resulting bicarbonate pool feed into the carboxysome – a bacterial protein-derived organelle that houses Rubisco and carbonic anhydrase within a semi-permeable protein shell (Kerfeld and Melnicki, 2016; Rae et al., 2013) – where CO_2_ and ribulose-bisphosphate react to form the central metabolite 3-phospoglycerate (3-PGA).

Carboxysomes are comprised of an outer protein shell and an inner enzymatic cargo, and their primary function is to (i) decrease photorespiration by avoiding high [O_2_] from reaching Rubisco (Li et al., 2020; Ferlez et al., 2019), (ii) concentrate CO_2_ around Rubisco >1000x (Badger and Price, 2003), and (iii) physically compartmentalize the cytosolic bicarbonate pool from the wide majority of carbonic anhydrase activity to prevent carbon loss (Cai et al., 2009; Dou et al., 2008; Price and Badger, 1989) (**Figure 1A**). Functional carboxysomes are essential for cyanobacterial growth at low (ambient) CO_2_ environments (Abernathy et al., 2019), therefore, we must fully understand their underpinning mechanisms for their ultimate manipulation as modules for carbon fixation. Structural features of cyanobacterial carboxysomes.

Carboxysomes are part of a larger class of protein-bounded organelles in bacteria called bacterial microcompartments (BMCs). All BMC shells, including those of carboxysomes, are built from an array of structurally conserved hexameric (BMC-H), pseudo-hexameric/trimeric (BMC-T), and pentameric (BMC-P) proteins (Kerfeld et al., 2018; Melnicki et al., 2021). These proteins natively self-assemble into icosahedral shells which form a barrier between the bacterial cytosol and the interior of the BMC. BMC-H proteins consist of a single Pfam00936 domain and, together with BMC-T and their various permutations (Sutter et al., 2021), make up the bulk of the facets by tessellating tightly into a honeycomb-like lattice (Sutter et al., 2016). BMC-P, on the other hand, consists of a Pfam03319 domain and exists more fleetingly within the shell (Yang et al., 2020; Sun et al., 2022; Sutter et al., 2017), serving to only cap the vertex positions (Cai et al., 2009; Sutter et al., 2017; Tanaka et al., 2008). All BMC shell proteins have characteristic concave (cytosol facing) and convex (luminally facing) surfaces (Sutter et al., 2017; Trettel et al., 2022). Together, these proteins assemble into a barrier that enables selective influx/efflux of metabolites (Dou et al., 2008) thanks to central pores located at their central axis of symmetry (Kerfeld et al., 2005).

Carboxysomes are categorized into 2-classes; α- and β-carboxysomes housed in α- and β-cyanobacterial lineages (using *cso* or *ccm* gene nomenclature), respectively. While structurally conserved, experimental evidence suggests that ɑ- and β-carboxysomes differ in their evolution, operon structure, components (Rubisco type, scaffolds, carbonic anhydrases), and modes of assembly (Kerfeld and Melnicki, 2016; Rae et al., 2013) (**Figures 1B, C**). Current models propose that ɑ-carboxysomes assemble concomitantly (Kerfeld and Melnicki, 2016; Iancu et al., 2010) with the disordered scaffold protein CsoS2 acting as an essential hub (Cai et al., 2015a) that supports the co-condensation of the Rubisco holoenzyme and carbonic anhydrase (Oltrogge et al., 2020; Blikstad et al., 2023) with mosaicked shell subunits (Ni et al., 2023), eventually maturing into a concentration-dependent paracrystalline/fibril array of Rubisco packaged within the shell (Ni et al., 2023; Metskas et al., 2022; Evans et al., 2023). In contrast, β-carboxysome assemble core-first (Cameron et al., 2013) with the essential scaffold protein CcmM initiating the condensation of Rubisco (Ludwig et al., 2000; Wang et al., 2019; Zang et al., 2021; Wang and Hayer-Hartl, 2023; Ryan et al., 2019), carbonic anhydrase (Long et al., 2007; Long et al., 2011), and CcmN into a ‘pro-carboxysome’. The encapsulation peptide (EP) of CcmN (Kinney et al., 2012) promotes shell envelopment of the pro-carboxysome resulting in a mature particle (Cameron et al., 2013; Chen et al., 2013) where Rubisco also exists in a paracrystalline lattice (Faulkner et al., 2017). Despite their functional differences, BMC particles [carboxysomes and metabolosomes (Yang et al., 2022)] rely on the liquid-liquid phase separation (LLPS) (Azaldegui et al., 2021) of their internal components to trigger their assembly. Despite recent experimental insights, engineering aspects such as size, morphology, and multiplexed cargo packaging remain a challenge.

## 3 Atomic-level description of shell permeability

**Figure 2:**
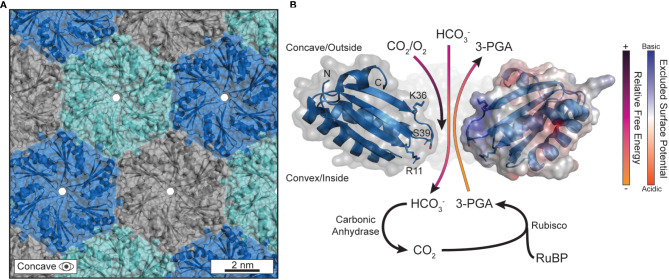
The pores in the protein shell are responsible for gating substrate permeation. **(A)** The outer protein shell is composed of hexagonally arranged shell proteins that tesselate into a tight-knit honeycomb-like lattice that forms a barrier against the cytosol. Currently, the only understood path for substrate diffusion is through pores that form at the central axis of symmetry in the hexameric quaternary structure. The PDB structure for CsoS1A (2G13) was used to generate this panel in PyMOL. **(B)** The biophysical properties that differentiate substrates, and their permeation through the central pore, are sequence encoded. Several substrates are visualized traversing the pore with their anticipated trajectories colored by their approximate relative free energy at that location as informed by Faulkner et al. The PDB structure for CcmK2 (2A1B) was used to generate this panel in PyMOL. The C-termini were clipped at residue 90 for clarity. The right-most monomer has its surface colored according to its excluded surface potential as calculated by the ABPS plugin in PyMOL.

Carboxysome shells enhance carbon assimilation by concentrating CO_2_ and limiting O_2_ diffusion within the luminal space while enabling the influx of bicarbonate and blocking CO_2_ leakage outwards (Rae et al., 2013; Cai et al., 2009; Dou et al., 2008). Permeation is understood to primarily occur at the central pores in the cyclic axis of symmetry in BMC-H shell proteins (Kerfeld et al., 2005) (**Figure 2A**). These pores, being typically ~4-7 Å in diameter (Tanaka et al., 2008; Kerfeld et al., 2005; Tanaka et al., 2009), have been experimentally attributed as gates for substrate passage. For example, mutagenized pore-adjacent residues on BMC-H proteins alter the biochemical activity for the entire BMC particle *in vitro* as well as cell growth when tested *in vivo* (Chowdhury et al., 2015); it is hypothesized that these mutations at the pore constriction change the rate at which the interior enzymes access substrates that diffuse through those pores.

### 3.1 The biophysical properties of the central pore present an energetic barrier to diffusion

MD approaches have affirmed and expanded upon the evidential importance of the central pore for substrate gating. Pioneering work came from modeling the major BMC-H protein (Yang et al., 2020) of the propanediol metabolosome in *Salmonella enterica*, PduA, using biased potentials (e.g. umbrella sampling, metadynamics) (Park et al., 2017). Calculations indicated a more favorable passage of 1,2-propanediol compared to the higher free-energy barrier for propionaldehyde (Park et al., 2017), confirming earlier assumptions that some intermediates, like the reactive propionaldehyde, are selectively sequestered within the BMC lumen to prevent toxicity (Sampson and Bobik, 2008) or carbon loss due to volatility (Penrod and Roth, 2006). The higher free-energy barrier does not wholly block substrates, like propionaldehyde, from diffusing but does impede the process kinetically. The mechanism was attributed simply to the higher hydrogen-bonding capacity of the central pore constriction, lined with a serine residue (S40) (Crowley et al., 2010), for 1,2-propanediol over propionaldehyde due to the extra hydroxyl group which acts as an additional hydrogen bond donor. Effectively, the serine-lined pore is a better binding site for 1,2-propanediol than propionaldehyde which promotes the formers passage so long as the binding is not overly strong (Bauer and Nadler, 2006). Such features from the Pdu BMC can reasonably be applied to carboxysomes, due to the high sequence and structural conservation between all BMC shell proteins (Melnicki et al., 2021). Indeed, experimental work in the propanediol metabolosome has further attributed the pore-lining S40 of PduA as critical to influencing permeation (Chowdhury et al., 2015) This result was later confirmed via simulation and experimentation on CcmK2 of the *Synechocystis* sp. PCC 6803 β-carboxysome with its S39 pore (Faulkner et al., 2020).

### 3.2 BMC-H surface electrostatics aid in substrate discrimination

The residues lining the central constriction alone are not sufficient to explain substrate permeation across ɑ- and β-carboxysome shells as many BMC-H proteins common encode residues like serine and glycine at the pore constriction. Surface electrostatic density around the central pore has also been observed to be an effective mechanism of attracting/repelling substrates in several studies focusing on BMC-H proteins (Faulkner et al., 2020; Mahinthichaichan et al., 2018). In all cases, the pore-adjacent concave surface exhibits a high extent of positive charge effectively turning the outward facing surface into an electrostatic funnel (**Figure 2B**). This may explain the lower free-energy profile for bicarbonate just outside of the pore within the concavity, essentially attracting the negatively charged bicarbonate anions while conversely impeding the passage of neutral CO_2_ and O_2_ from reaching the interior [or, in the case of CO_2_, escaping once bicarbonate is converted luminally by carbonic anhydrase (Cai et al., 2009)] (**Figure 2B**). These works importantly provided the groundwork to understand not just CO_2_ concentration within the shell, but also the inhibition of O_2_ diffusion too. MD observations that O_2_ diffusion is biophysically impeded is substantiated by the presence of bioinformatically identified glycl-radical enzyme associated microcompartments (GRMs) (Zarzycki et al., 2015). GRMs have been speculated to help extend the range of environments that glycyl-radical enzymes can reasonably act in, as oxygen exposure inactivates these enzymes (Zarzycki et al., 2017; Zhang et al., 2001). This notion is further supported by engineered carboxysome shells that package oxygen-sensitive hydrogenases can impart enhanced activity in an aerobic environment (Li et al., 2020).

The specific residues (corresponding to R11, K36, and the pore S39 in CcmK2) responsible for forming a substrate barrier are largely conserved among CcmK2 proteins (Faulkner et al., 2020) and emphasize the importance of both the pore and concave surface overall in substrate gating (**Figure 2B**). The alignment of β-strands proximal to the pore on the concave surface (L31 to K36 in CcmK2) also expose their backbone amines and contribute to this effect. We note, however, that these specifics will differ between shell proteins. For example, BMC-H even among the same class will differ in surface electrostatics (Schmidt-Dannert et al., 2018) and therefore should not be taken as a one-size-fits-all rule (i.e. concave surface always being positive to the same degree) but instead as another layer of consideration when assessing permeation. While Rubisco and the carboxysome shell may not be able to sufficiently differentiate between CO_2_ and O_2_ (Poudel et al., 2020), the outer shell can enhance the passage bicarbonate and, in combination with the encapsulated carbonic anhydrase, locally increase the CO_2_ concentration around Rubisco. MD simulations have helped explain the molecular basis for carbon concentration in the carboxysomal CCM and will be an essential methodology to predictively modify the shell for augmented substrate specificities moving forward.

### 3.3 Competition for pore occupancy may regulate permeation events

The proclivity of BMC shell pores to bind anionic species is not limited to solely bicarbonate. Other anions, like sulfate, have been found in crystal structures of a broad range of BMC-H proteins including CcmK1, CsoS1A, and EutM to list a few (Tanaka et al., 2008; Tsai et al., 2007; Takenoya et al., 2010). MD have also revealed structural aspects of ion coordination. For instance, chloride ions have been observed to occupy pore-adjacent positions (Faulkner et al., 2020; Mahinthichaichan et al., 2018) and coordinate with either backbone amides or basic residues, such as arginine. Similar results were recently found for the metabolosome BMC-H PduA, where chloride ions were found to coordinate with the backbone amide of the pore-lining S40 (Trettel et al., 2023). This study also found that chloride itself also acts competitively with 1,2-propanediol, the intended substrate, for pore access thereby hindering permeation rates. Altogether, simulation data from both carboxysome and metabolosome models both agree on the ability of anions to coordinate with and occupy shell protein pores via non-specific backbone interactions (Faulkner et al., 2020; Trettel et al., 2023). While only currently reported for metabolosome shells, this suggests that ion coordination may be a widespread phenomenon which can also regulate permeation events in carboxysomes. The role of other physiologically relevant anions, such as inorganic phosphate which can regulate Rubisco activity (Marcus and Gurevitz, 2000), has yet to be explored in this context.

## 4 Modeling the physical principles underlying carboxysome assembly

Bacterial microcompartments, including carboxysomes, can vary in size and regularly do not demonstrate a singular defined structure. This differs greatly from similarly icosahedral, although evolutionarily unrelated (Krupovic and Koonin, 2017), viral capsids and complicates the direct structural assessment of native BMC complexes. Understanding the dynamics of carboxysome self-assembly can shed light on the polydispersity and factors that control it and thereby tune factors which directly contribute to carbon fixation like surface-to-volume ratios, Rubisco organization, and Rubisco packaging efficiency. While inspired by simulations that explain viral capsid assembly that typically form around nucleic acids (Perlmutter et al., 2013; Lynch et al., 2023), new models pertaining to BMC assembly specifically needed to be developed to explain the subtle differences that trigger biogenesis and heterogeneous assemblies.

### 4.1 Cargo interactions are the differentiating factor between assembly pathways

**Figure 3:**
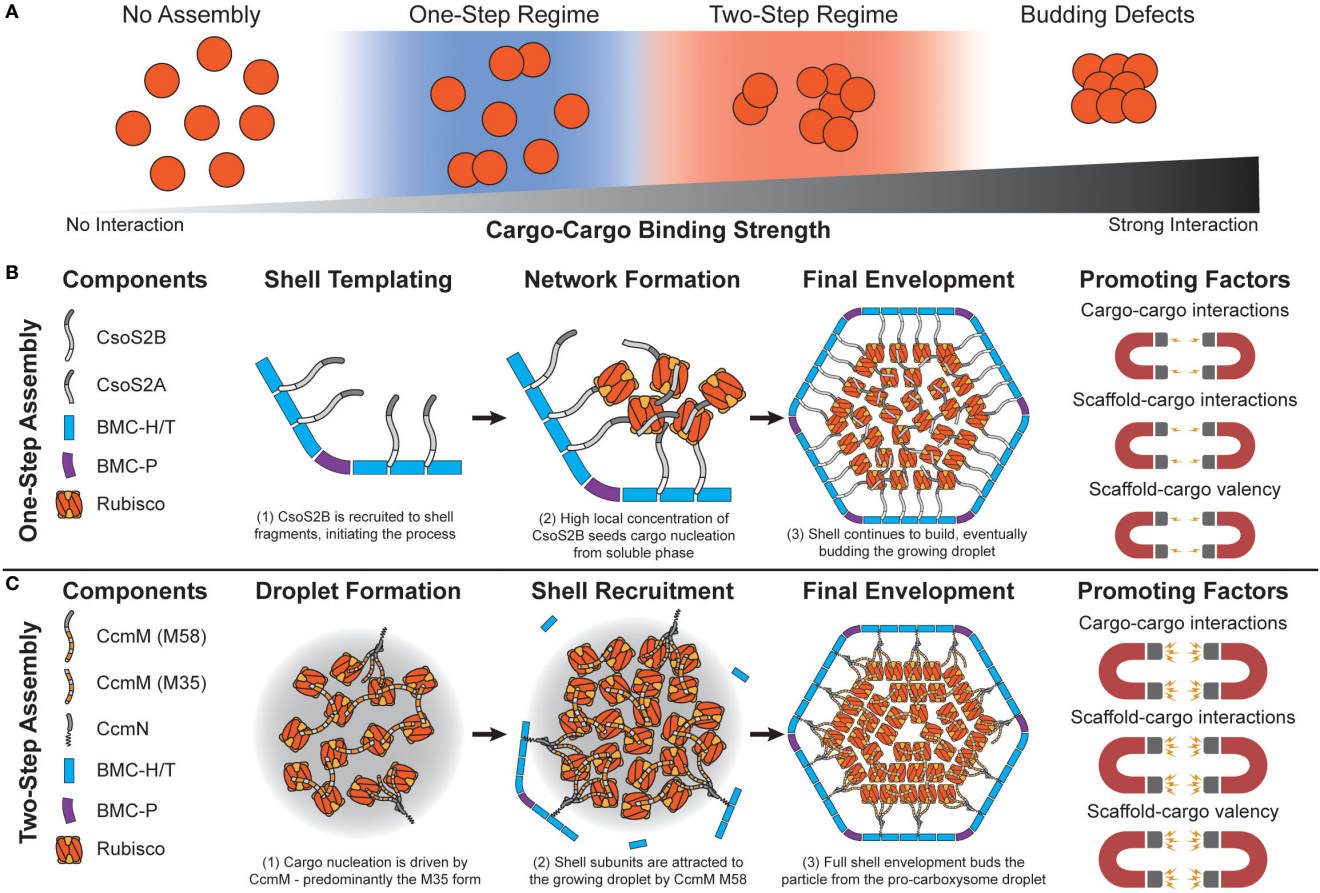
Carboxysome assembly pathways depend on the relative propensity of cargo to aggregate. **(A)** Simulations predict that carboxysome assembly pathway exists on a continuum dependent on cargo-cargo binding strengths. Very weak or no binding propensity inhibits assembly of filled shells. Weak/moderate binding strengths results in one-step assembly pathways, as either high concentrations of cargo or shell components that locally increase cargo concentration are needed. Moderate/strong binding strengths lead to a two-step pathway, where cargo can coalesce independent of a shell. Overly strong binding strengths inhibits budding of the cargo droplet by shell components. **(B)** In the one-step pathway (also called concamitant), cargo proteins Rubisco, CsoS2A, and CsoS2B do not interact strongly enough to inititate phase separation from the bulk. CsoS2B must first bind shell facets/vertices. This creates a local environment with a high concentration of CsoS2B N-terminal repeats that attract Rubisco and CsoS2A. The droplet growth cascades until a critical mass of shell proteins envelope it, resulting in a mature alpha carboxysome. Molecular simulations reveal that this pathway is promoted by relatively weaker cargo-cargo interactions/valency. **(C)** In the two-step pathway (also called core-first), cargo proteins Rubisco and both forms of CcmM together coalesce a pro-carboxysome droplet. CcmN allows for shell components to begin templating around the growing droplet, eventually budding a complete particle. Molecular simulations reveal that this pathway is promoted by relatively strong cargo-cargo interactions/valencies. The carbonic anhydrase component in both examples is omitted for clarity.

**Figure 4:**
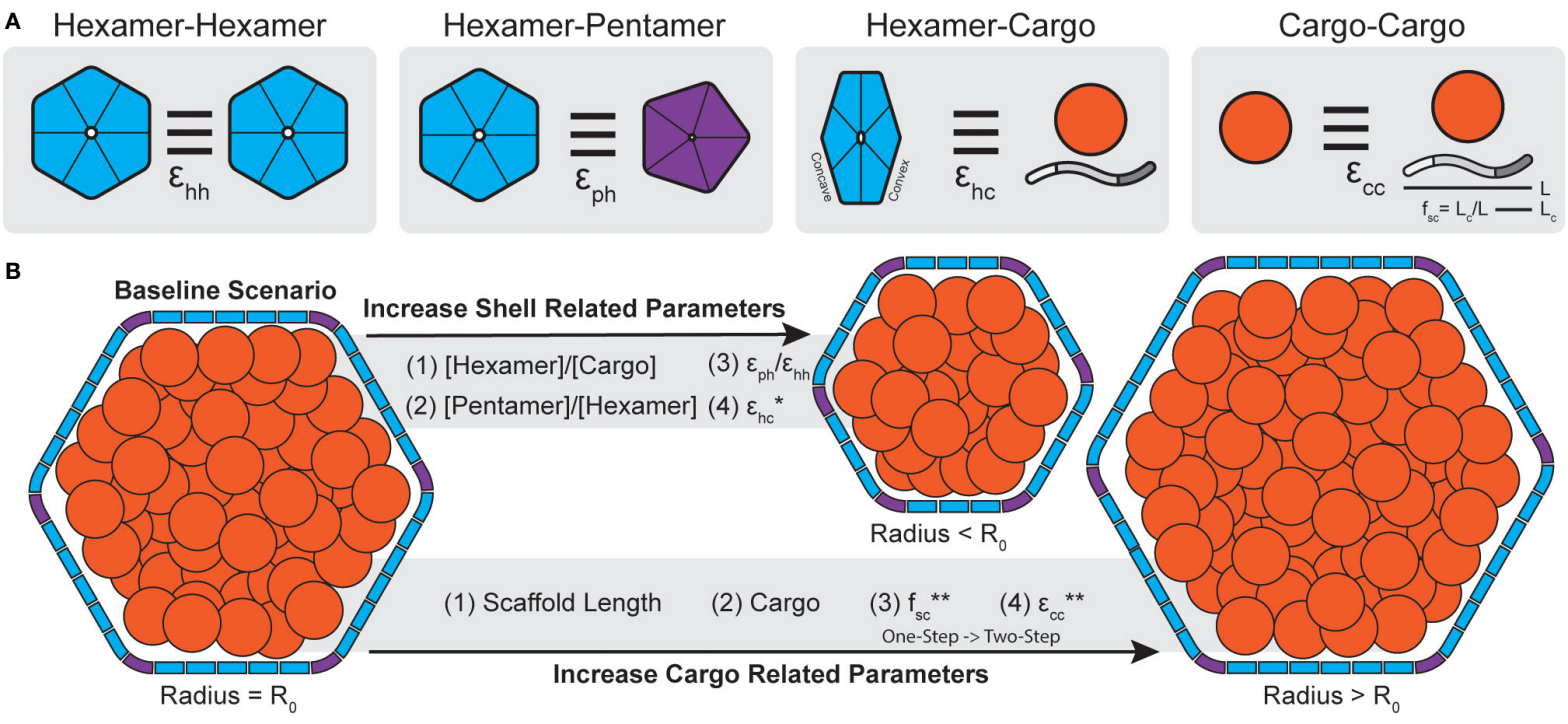
Carboxysome morphology is determined by a combination of shell and cargo related parameters. **(A)** The interactions strengths between different components of a BMC can be parameterized (ϵ). In simulations, hexamers can interact edge-to-edge while having matching surfaces oriented in parallel with interactions strength ϵ_hh_. Hexamers and pentamers can likewise interact with strength ϵ_ph_. Cargo components, defined as both scaffolds like CsoS2 (shown) or general cargo (orange circle) can interact with the convex surface of shell hexamers with strength ϵhc. Lastly, cargo, in the form of general cargo or scaffolds, can form self interactions of strength ϵ_cc_. In the case of scaffolds, the fraction of the protein that bind cargo (f_sc_) is defined as the length of the cargo binding domain (L_c_, akin to valency or number of binding sites) divided by the total scaffold length (L). **(B)** Molecular simulations reveal parameters that alter morphology during assembly. Generally, increasing parameters (stoichiometry, interaction strengths) related to the shell lead to smaller particles. Conversely, increasing parameters related to cargo aggregation, or including cargo at all, leads to larger particles. One except is that increasing ϵ_hc_ for general cargo will result in smaller particles but for scaffolds will not (*, see Mohajerani et al., 2021). Note, assembly pathways in simulations shift from one-step to two-step with increasing f_sc_ and e_cc_ (**). Accordingly, the two-step pathways are generally associated with larger particles.

Initial attempts at modeling BMC assembly were inspired by carboxysomes where evidence has been found for both concomitant and core-first assembly pathways (Perlmutter et al., 2016) as observed in both ɑ- and β- lineages (Kerfeld and Melnicki, 2016) (**Figure 3**). The principle differentiating factor was the relative strength of attraction cargo had for other cargo, where weaker interactions led to ‘one-step’ or concomitant assembly (observed in ɑ-carboxysomes) (**Figures 3A, B**) while stronger interactions led to ‘two-step’ or core-first (observed in β-carboxysomes) (**Figures 3A, C**). Specifically, for ɑ-carboxysomes, modeling (Mahalik et al., 2016) and atomic-force microscopy (Sutter et al., 2016; Garcia-Alles et al., 2017) have both suggested that shell facets form by nucleation, which can further provide an area to locally concentrate cargo (Oltrogge et al., 2020) and nucleate ɑ-carboxysome formation, since cargo-cargo interactions are predicted to not be strong enough drivers on their own (Perlmutter et al., 2016) (**Figure 3B**). β-carboxysome cargo (Rubisco and CcmM M35) in two-step assembly modes coalesce strongly enough on their own without the need of a shell-templated trigger (**Figure 3C**). Interestingly, these simulations predicted that cargo would become organized into concentric layers, observed prior in both ɑ- (Iancu et al., 2007; Shively et al., 1973; Schmid et al., 2006) and β-carboxysomes (Kaneko et al., 2006; Iancu et al., 2005). Paracrystalline order was not a prerequisite for forming complete particles in these simulations and in fact would inhibit budding (**Figure 3A**). These observations have held up to additional recent higher-resolution experimental scrutiny, where Rubisco in both ɑ- and β-carboxysomes is now understood to assemble into concentric layers (Evans et al., 2023; Faulkner et al., 2017; Ni et al., 2022) when the internal concentration is sufficiently high (Metskas et al., 2022). While just the first of many follow-up studies, Perlmutter et al.’s above work demonstrated the utility of computational modeling to understand carboxysome assembly. However, the system employed at the time, albeit elegant, only investigated one shell geometry (T = 3), one BMC-H and BMC-P, and one cargo. This initial model has been greatly expanded to include considerations like the impact of cargo packaging on BMC size (Mohajerani and Hagan, 2018), the role of scaffolds (Mohajerani et al., 2021), and even multiple cargos (Tsidilkovski et al., 2022) on microcompartment size, assembly pathway, and packaging efficiency (**Figure 4A**).

In the context of microcompartment size, dynamical simulations reveal that shells packaged with cargo, generally, tend to be larger than non-packaged shells (Mohajerani and Hagan, 2018). Further, these simulations showed that BMC size also correlates with assembly pathways where core-first assembly modes, dominated kinetically by relatively stronger cargo-cargo interactions, led to larger particles and up to 5-fold more packaged cargo than concomitant modes (Mohajerani and Hagan, 2018). These results similarly apply to multi-component systems, where assembly pathway is primarily delineated by the sum of the cargo interaction strengths and the strength of self-cargo interactions (**Figure 4B**) can dictate packaging stoichiometry (Tsidilkovski et al., 2022). This has been likewise observed in real BMC systems where β-carboxysomes, which follow a core-first assembly pathway, tend to be larger than their ɑ- counterparts (Whitehead et al., 2014). Empty, synthetic structural models reported thus far are also always far smaller than native BMCs (<40 nm diameter) (Sutter et al., 2017; Ni et al., 2023; Tan et al., 2021; Sutter et al., 2019a; Kalnins et al., 2020; Greber et al., 2019; Sutter et al., 2019b).

### 4.2 The role of shell components in BMC assembly and morphology

Dynamical simulations have also revealed that shell components, while not the predominant factor, can also influence final morphology. For instance, simply increasing the ratio of shell proteins to cargo can lead to overnucleation (Mohajerani and Hagan, 2018) and thus smaller particles (**Figure 4B**). This has also been found in simulations which assume shell proteins demonstrate no spontaneous curvature of their own [motivated by atomic force microscopy studies on shell subunits (Sutter et al., 2016; Garcia-Alles et al., 2017)] and can essentially trap a growing cargo droplet out of equilibrium (Rotskoff and Geissler, 2018). This, however, may depend on the system of study as shell proteins have been observed to form sheets, nanotubes, and empty icosahedra among other morphologies, sometimes within the same sample (Ferlez et al., 2023), without the need of cargo templating to induce curvature (Ferlez et al., 2023; Trettel and Winkler, 2023; Uddin et al., 2018; Hagen AR. et al., 2018; Noël et al., 2015). The presence of excess pentamers or stronger pentamer-hexamer interactions can likewise lead to more pentamer insertion and thus overnucleation into smaller particles (Mohajerani and Hagan, 2018) (**Figure 4B**). This latter point is interesting since many BMC operons encode for more than one BMC-P (Sutter et al., 2021). BMC-P proteins appear to play different roles in different contexts, where in some metabolosomes they can directly influence BMC morphology (Mills et al., 2022) and in others they are completely dispensable and can be added exogenously to “cap” the icosahedron (Sutter et al., 2019b; Hagen A. et al., 2018; Kirst et al., 2022). Observations from simulations further emphasize the importance of studying the effects of BMC-P and how they can influence morphology, packaging efficiency and permeability.

### 4.3 The role of carboxysome-inspired scaffolds in BMC assembly

The above studies ascribed the connection of homogenous cargo to end morphology. However, the models used may be more applicable to metabolosomes, where cargo directly interacts with the shell (Fan et al., 2010; Aussignargues et al., 2015), than carboxysomes, where scaffolds act as an intermediary connecting the shell and cargo domains (Oltrogge et al., 2020; Wang et al., 2019). Accordingly, Mohajerani et al. have also conducted a study, motivated by ɑ-carboxysomes specifically, on the role of a CsoS2-inspired scaffold proteins in BMC assembly (Mohajerani et al., 2021) (**Figure 4A**). Scaffolds proteins, as a type of cargo themselves, can potentially affect shell size and assembly pathway in a much more programmable manner than typical cargo due to their modular nature (Chaijarasphong et al., 2016) (**Figure 1C**). Simulations parametrized these physical aspects by defining the length of the CsoS2-inspired scaffold (L), the length of the cargo binding domain [L_c_, with longer L_c_ meaning more cargo binding sites, akin to more CsoS2 N-terminal domains (NTDs)] and the fractional length of the cargo binding domain (f_sc_ = L_c_/L) as shown in **Figure 4A**. Importantly, simulations reveal that there is a critical interplay between the total length of the scaffold and its valency with cargo. By fixing the overall scaffold length (L) and increasing L_c_ (and therefore f_sc_), simulations showed that cargo packaging likewise increases. Moreover, increasing f_sc_, analogous to the number of cargo binding sites, transitioned systems from a one-step to a two-step assembly pathway (**Figure 4B**) where two-step pathways are again associated with more cargo packaging (Mohajerani et al., 2021). Similarly to cargo packaging alone (Mohajerani and Hagan, 2018), as the scaffold is itself a type of cargo, physically longer scaffolds also generally result in larger shells to a point as they increase volume requirements (Mohajerani et al., 2021). These simulations are supported by work in the model *H. neapolitanus* α-carboxysome that demonstrates a requirement for a minimal threshold of NTDs in CsoS2 to be met to achieve carboxysome formation (Oltrogge et al., 2020). Further, more recent work by Oltrogge and colleagues likewise agree that increasing CsoS2 length by increasing the number of middle region (MR) repeats leads to larger α-carboxysomes (Oltrogge et al., 2023). However, they ascribe this phenomenon to the MR repeats of CsoS2 stabilizing the low-curvature regions (i.e. the facets) of the carboxysome shell, enabling their extension, while Mohajerani et al. argue for the need to meet increased volume requirements. We note that these arguments are not mutually exclusive.

## 5 Discussion

### 5.1 Areas for growth in understanding permeation

MD simulations of shell permeation to date have focused on a small subset of model BMC-H. While impactful, future permeation studies may wish to sample a greater diversity of BMC-H to develop a deeper understanding of the natural biophysical diversity shell proteins can accommodate. For instance, sampling a wider array of carboxysomal BMC-H may highlight subtle differences that influence bicarbonate, O_2_, and 3-PGA diffusion. Similar methodologies can and should be applied towards describing permeation in mixed heterohexamer systems, like those reported for CcmK3/K4 (Sommer et al., 2019; Garcia-Alles et al., 2019) or purely synthetic systems with the potential for asymmetric pore designs (Česle et al., 2023) that may further regulate substrate diffusion in ways that homo-hexameric BMC-H cannot. The various classes of BMC-T should also be considered to better grasp their hypothesized connection to substrate gating (Klein et al., 2009; Tanaka et al., 2010). Simulation scale also needs to be accounted for, and future studies may wish to engage with physiologically relevant systems with multiple components like the small synthetically-derived BMC shells (Sutter et al., 2017; Ni et al., 2023; Tan et al., 2021; Sutter et al., 2019a; Kalnins et al., 2020; Greber et al., 2019; Sutter et al., 2019b), as a proxy for larger native-like systems. Investigating more complex shells will progress our understanding of how chemical gradients, a physiologically critical component, behave and evolve within a BMC context. For instance, differences in density and packing of Rubisco within carboxysomes (Kaneko et al., 2006; Ni et al., 2022) may result in CO_2_/O_2_ gradients proportional to the enzymes’ proximity to the shell. Detailed permeation studies could discern the packaging attributes within BMCs that would result in more efficient catalytic properties in engineered architectures. Simulating whole-BMC shell models can also limit pore-centric bias and explore if flux exists in non-porous areas such as the hexamer-hexamer interfaces or corner junctions where three hexamers meet. Similarly, permeability studies can be expanded to study the diffusion of a wider swath of metabolites and cofactors through BMC shell structures. Current research suggests that these cofactors, like NAD(P)H, are maintained as private pools that are internally recycled and do not appreciably diffuse through the shell barrier (Huseby and Roth, 2013; Cheng et al., 2012). Regardless, novel BMCs may be sought to transform metabolites far larger than those found in current model systems. MD simulations of permeation, therefore, will continue to facilitate rapid *in silico* prototyping of permeation through protein shells for altered substrate specificities or enhanced carbon concentration within the carboxysome lumen.

Specific structural components of the shell, such as the extended C-termini on many BMC-H, should also be addressed. While typically ignored due to missing crystallographic data, these termini can now be predicted and integrated into computational models thanks to emerging computational tools. These outward-facing, flexible/disordered (Faulkner et al., 2020) termini have been implicated in functions such as assembly (Trettel et al., 2022; Klein et al., 2009) like in viruses (Xue et al., 2014), but some data suggests they also reach into the concavity of adjacent subunits (Trettel et al., 2022) which may impact permeation or fine-tune assembly in environmentally responsive ways.

### 5.2 Future directions for studying carboxysome assembly

The collective knowledge on carboxysome systems continually expands and reinvents our understandings of these complex systems. Incorporating simulations to complement emerging experimental insights will lead to more meaningful outputs to inform design choices. For instance, future modeling may wish to explore evidence-informed shell-cargo interaction sites that form from predominantly (i) the edge-edge interaction surface of two adjoining shell proteins (Ni et al., 2023) and, in some cases, (ii) interactions with specific interior-oriented domains such as the N-terminus of the PduB BMC-T (Trettel et al., 2022; Lehman et al., 2017; Kennedy et al., 2022). This is further underpinned by the multitude of different shell proteins BMCs can encode and their synthetic interchangeability (Cai et al., 2015b; Slininger Lee et al., 2017) which certainly influence shell-shell (including curvature) and shell-cargo/scaffold interactions. For example, many BMCs encode for BMC-T proteins where every other edge may be better attuned for specific shell interactions on adjacent subunits (Trettel et al., 2022; Waltmann et al., 2023) and influence factors like shell curvature and/or shell-cargo interactions. Many others bioinformatically identified BMC loci entirely lack these factors for unknown reasons (Sutter et al., 2021).

The luminal organization of Rubisco is also now known to differ between related carboxysomes and may be tied to overall carboxysome activity, For instance, *Halothiobacillus* α-carboxysomes exhibit ~2-fold higher activity than *Cyanobium* α-carboxysomes and have different modes of Rubisco organization (Ni et al., 2022). Future simulations may have an opportunity to explain how these subtle structural differences arise (i.e. Rubisco surface charge difference, CsoS2 binding affinity, internal Rubisco concentration), ascribe functional consequences, and reveal how to program desired internal conformations.

Shell-focused assembly simulations can also help better define and explain the mechanisms behind the varied supramolecular structures BMC shell proteins can form *in vivo* and *in vitro*, such as nanotubes, for designer protein scaffolds (Young et al., 2017). Recent work suggests that BMC-H curvature trends can be inferred by their crystal structural arrangements (Garcia-Alles et al., 2023) and that these trends can be modulated rationally with computationally-informed amino acid substitutions (Li et al., 2021). However, factors like buffer/environmental composition (Faulkner et al., 2019), shell protein class and stoichiometry, and protein disorder undoubtedly also factor into supramolecular, and native-like, structures in unknown ways. In particular, the disordered termini many BMC-H proteins carry, predominantly on their outward facing C-terminus (Sutter et al., 2017; Trettel et al., 2022) have been speculated to fine-tune both shell-shell and shell-cargo interactions (Fan et al., 2012). Further, currently described simulation systems may already be attuned to ascribe the role of the multiple pentamers BMCs can encode for by tuning their relative stoichiometry and interaction strengths. Computational studies will undoubtedly continue to address these considerations and many more for custom carbon-fixing scaffolds.

### 5.3 Lessons for experimental carboxysome modifications

Assembly-focused simulations teach us that assembly pathway is chiefly governed by cargo interaction strengths (**Figure 3A**) while final morphology is determined by both cargo and shell contributions (**Figure 4B**). In terms of assembly pathway, stronger cargo-cargo (including scaffold) interactions or higher cargo stoichiometries are typically associated with two-step assembly pathways that lead to larger shells with more cargo (Mohajerani and Hagan, 2018; Mohajerani et al., 2021; Tsidilkovski et al., 2022) (**Figure 4B**). Conversely, weaker cargo-cargo interactions or higher shell stoichiometries are associated with one-step assembly pathways and smaller shells (Mohajerani and Hagan, 2018; Mohajerani et al., 2021; Tsidilkovski et al., 2022) (**Figure 4B**). These findings carry direct carboxysome design implications related to assembly kinetics that manifest physically in the forms of (i) expression system design and (ii) scaffold design.

Cargo and shell constructs can be designed in both a continuous synthetic operon or discontinuously into different plasmids with different modes of induction for testing (Lee and Tullman-Ercek, 2017). Single-vector/operon designs have been successful using a variety of induction approaches (Bonacci et al., 2012; Graf et al., 2018; Flamholz et al., 2020; Jiang et al., 2023). Notably, similar strategies also result in morphologically and functionally sound carboxysomes when genomically integrated and expressed in plants (Chen et al., 2023; Long et al., 2018). Double-vector systems, which independently express shell and cargo components, have also been described (Jiang et al., 2023; Jakobson et al., 2016; Wagner et al., 2017) although they do need to be tuned and timed appropriately (Lee and Tullman-Ercek, 2017; Jakobson et al., 2016; Nichols et al., 2019) likely due to kinetic effects of aggregation described by simulations. In one case, researchers redesigned a carboxysome for hydrogen production by serially inducing hydrogenase cargo followed by a β-carboxysome shell (Li et al., 2020). BMCs with concomitant assembly pathways, like those commonly employed for heterologous α-carboxysomes formation, may benefit from single vector designs which promote co-expression of both shell and cargo components under native-like controls (i.e. ribosomal binding sites). Similarly, two-step pathways may be promoted by a well-tuned cargo-preaggregation step proceeded by shell expression. Researchers should consider the kinetics of interactions and expression to prevent off-target assemblies.

Scaffold choice and design is also an emerging route for modification. In ɑ-carboxysomes, modifying CsoS2 and the ratios of CsoS2A and CsoS2B (analogous to f_sc_ in simulations) or the number of NTD/MR repeats (Oltrogge et al., 2023) are approachable routes to alter morphology and Rubisco packaging for CCM augmentation. Similarly, in β-carboxysomes, modifications of CcmM and CcmN may also be sufficient routes for modification. However, both classes of scaffolds act as specific adaptors between the Rubisco cargo and the shell domains and therefore cannot coalesce a more diverse range of cargo by themselves without extensive modification. Heterologous encapsulation and assembly methods may wish to rely on carboxysome-inspired fusions (Gonzalez-Esquer et al., 2015) or metabolosome EPs which trigger both shell-cargo (Fan et al., 2010) and cargo-cargo (Lawrence et al., 2014). Cargo fused with metabolosome EPs may act more akin to the assembly models produced in several assembly simulation works to date (Perlmutter et al., 2016; Mohajerani and Hagan, 2018; Tsidilkovski et al., 2022).

One bottleneck with biomass productivity lies in the connection between photosynthetic efficiency and carbon fixation. To alleviate these bottlenecks, some groups have installed various CCM components into plant chloroplasts including *Nicotiana benthamiana* (Lin et al., 2014), *Rhodosprillum rubrum* (Long et al., 2018), and *Nicotiana tabacum* (Chen et al., 2023). These studies have been able to generate carboxysomes nearly structurally and catalytically equivalent to native carboxysomes and support photosynthesis (Chen et al., 2023). Further additional factors like the incorporation of bicarbonate transporters, removal of the stromal carbonic anhydrase, and including Rubisco activates (Chen et al., 2022) may be needed to significantly enhance growth under ambient CO_2_ conditions. A deeper fundamental understanding of carboxysome assembly offered by computational simulations may assist in full implementation of cyanobacterial CCMs into C3 plants.

### 5.4 The future of computational models and methods

Simulations have played a critical role in exploring the physical phenomena that underpin carboxysome assembly. However, many conclusions remain explored at low resolution, partially due to the technics used. More investment in multi-resolution calculations is required for incorporating high accuracy detailed mechanisms at commensurate computational investment. Such methodologies may only be possible after exercising high fidelity energy landscape reconstruction based on accelerated MD or AI assisted methodologies for the fast interconversion between low resolution models (e.g. supra coarse-grained) and fully atomic detailed structures. Only this approach would be able to lead to a more fine-tuned and robust rational carboxysome manipulation.

## 6 Conclusion

The integration of *in silico* predictive and analytical methods with *in vivo* structure/function studies of BMCs is essential to advance BMC-based biotechnologies. MD simulations have been critical in describing the fundamental principles underlying permeation events through protein shells and fundamental principles that underpin carboxysome assembly. MD simulations reveal that substrate permeation is controlled by a series of biophysical properties, encoded by residues mainly along the outer concave surface, and substrate competition. Simulations studying BMC assembly demonstrate that assembly pathway is controlled kinetically by cargo accumulation and morphology is dictated by a combination of shell and cargo parameters. The ever-increasing access to computational power, and methodologies (i.e., machine-learning algorithms), will undoubtably expand these findings and allow for a higher-throughput exploration of the BMC diversity and the redesign of these architectures for specific non-native biochemical traits. Such advancements will continue to impact how we think and tinker with these architectures and help implement programmable BMCs for biomanufacturing and enhanced CO_2_ sequestration roles.

## Author contributions

DT: Conceptualization, Formal Analysis, Investigation, Writing – original draft, Writing – review & editing. SP: Writing – original draft, Writing – review & editing. AL: Writing – original draft, Writing – review & editing. CG: Conceptualization, Funding acquisition, Project administration, Supervision, Writing – original draft, Writing – review & editing.
